# Screening of antibacterial compounds with novel structure from the FDA approved drugs using machine learning methods

**DOI:** 10.18632/aging.203887

**Published:** 2022-02-12

**Authors:** Wen-Xing Li, Xin Tong, Peng-Peng Yang, Yang Zheng, Ji-Hao Liang, Gong-Hua Li, Dahai Liu, Dao-Gang Guan, Shao-Xing Dai

**Affiliations:** 1 Department of Biochemistry and Molecular Biology, School of Basic Medical Sciences, Southern Medical University, Guangzhou 510515, Guangdong, China; 2 Guangdong Provincial Key Laboratory of Single Cell Technology and Application, Southern Medical University, Guangzhou 510515, Guangdong, China; 3 State Key Laboratory of Primate Biomedical Research, Institute of Primate Translational Medicine, Kunming University of Science and Technology, Kunming 650500, Yunnan, China; 4 State Key Laboratory of Genetic Resources and Evolution, Kunming Institute of Zoology, Chinese Academy of Sciences, Kunming 650223, Yunnan, China; 5 School of Medicine, Foshan University, Foshan 528000, Guangdong, China

**Keywords:** antibacterial compound, drug repositioning, machine learning, structural similarity, virtual screening

## Abstract

Bacterial infection is one of the most important factors affecting the human life span. Elderly people are more harmed by bacterial infections due to their deficits in immunity. Because of the lack of new antibiotics in recent years, bacterial resistance has increasingly become a serious problem globally. In this study, an antibacterial compound predictor was constructed using the support vector machines and random forest methods and the data of the active and inactive antibacterial compounds from the ChEMBL database. The results showed that both models have excellent prediction performance (mean accuracy >0.9 and mean AUC >0.9 for the two models). We used the predictor to screen potential antibacterial compounds from FDA-approved drugs in the DrugBank database. The screening results showed that 1087 small-molecule drugs have potential antibacterial activity and 154 of them are FDA-approved antibacterial drugs, which accounts for 76.2% of the approved antibacterial drugs collected in this study. Through molecular fingerprint similarity analysis and common substructure analysis, we screened 8 predicted antibacterial small-molecule compounds with novel structures compared with known antibacterial drugs, and 5 of them are widely used in the treatment of various tumors. This study provides a new insight for predicting antibacterial compounds by using approved drugs, the predicted compounds might be used to treat bacterial infections and extend lifespan.

## INTRODUCTION

Due to deficits in innate immunity and adaptive immunity in older adults, they are more susceptible to viral and bacterial infection and experience higher incidence and severity of infectious diseases [1]. Bacterial infections may also cause common neurodegenerative disorders [2], such as Alzheimer’s disease [3]. Antibiotics are a fast and effective way to deal with bacterial infections. However, with the widespread use of antibiotics, bacteria are also constantly evolving and a large number of pathogens have emerged that can resist these drugs [4]. As the research and development of novel antibiotics by pharmaceutical companies has drastically decreased in recent years, bacterial resistance has increasingly become a serious problem [5]. Therefore, the development of novel and highly efficient antibiotics is an urgent issue. High-throughput screening has been the dominant approach of antimicrobial drug development in the industry in the past few decades [6, 7]. However, due to the long development time, huge cost, and low efficiency of this method [8], computer-aided drug design techniques have become a promising method in the discovery of novel antibacterial drugs [9].

Previous studies have developed multiple computational methods for efficiently assessing and screening compounds for their antimicrobial activity, such as multitarget and multi-objective approaches [10, 11]. Quantitative structure-activity relationships (QSAR) modeling is one of the most frequently employed *in silico* techniques for antibacterial activity prediction, improved models such as mt-QSAR and QSAR-Co can integrate multi-dimensionally heterogeneous chemical and biological data which greatly improved the reliability of such modeling [12]. The current view holds that drugs are inherently poly-pharmacological because they can act on multiple targets or disease pathways, and thus the drug discovery process should attempt to optimize more properties simultaneously [13]. Based on this theory, the multi-task model constructed by comprehensively considering the antibacterial activity of the compound and ADMET (absorption, distribution, metabolism, excretion, toxicity) characteristics can accurately screen anti-mycobacterial drugs [14]. Molecular fingerprints are a way of encoding molecular structure that digitizes the structural information of a compound, which are widely used in drug discovery and virtual screening [15]. Compared to other virtual screening methods, molecular fingerprints require minimal setup and configuration, are easy to calculate, and are less CPU-intensive and memory-intensive, which become the preferred tool for characterizing small molecules [16].

In recent years, machine learning methods have shown tremendous potential in the process of drug discovery and development [17]. Multiple machine learning-based methods effectively improved the accuracy of drug-target interaction prediction [18]. Especially in the early phases of drug discovery, the use of machine learning methods significantly reduces time and effort in drug discovery and development [19]. In other areas of drug discovery, deep learning is a promising method for the prediction of molecular properties and the de novo generation of suggestions for new molecules [20]. Compared with traditional methods, machine learning approaches have the advantages of high precision, low cost, and strong operability. These technologies may have fundamentally changed the process of identifying new molecules and/or repurposing old drugs [21]. Multiple machine learning methods are widely used in ligand-based and receptor-based antibacterial drug discovery [9, 22–27]. By using chemoinformatics methods to extract the molecular characteristics of short peptides, studies have shown that the support vector machine (SVM) model can accurately predict the antibacterial activity of short peptides [22, 23] and the genetic characteristics of antibiotic resistance in specific pathogens [28]. The combination using random forest (RF) and genome-based analysis approaches promoted phenotypic antibacterial drug discovery [24] and revealed potential antibiotic resistance genes [25]. In recent years, emerging deep neural network methods have facilitated the discovery of antibacterial molecules with unique structures from massive data [26]. Furthermore, due to the limitations of a single method, the combination using multiple machine learning methods showed excellent performance in antibacterial compounds discovery [26, 27] and predicting the bacterial genetic mutations on drug resistance [29].

Although the popularization of machine learning methods has greatly shortened the discovery of antibacterial lead compounds, there are still required long-term studies from the identified lead compounds to clinical applications, especially experiment on drug safety [30]. Therefore, a new use for old drugs may be a way to resolve current antibiotic resistance [31]. The current Food and Drug Administration (FDA) approved antibiotics can be divided into multiple categories according to the core scaffolds, and a variety of semi-synthetic antibiotics are based on these scaffolds [32]. Due to the increased bacterial resistance to specific scaffold structures, it is a promising way to develop antibiotics with novel structures. In this study, we combined using multiple machine learning methods and molecular fingerprints of compounds to build the antibacterial compound predictor and then identified structure novel small-molecule antibacterial compounds from the FDA-approved drugs.

## RESULTS

### Initial screening of machine learning methods

**Figure 1:**
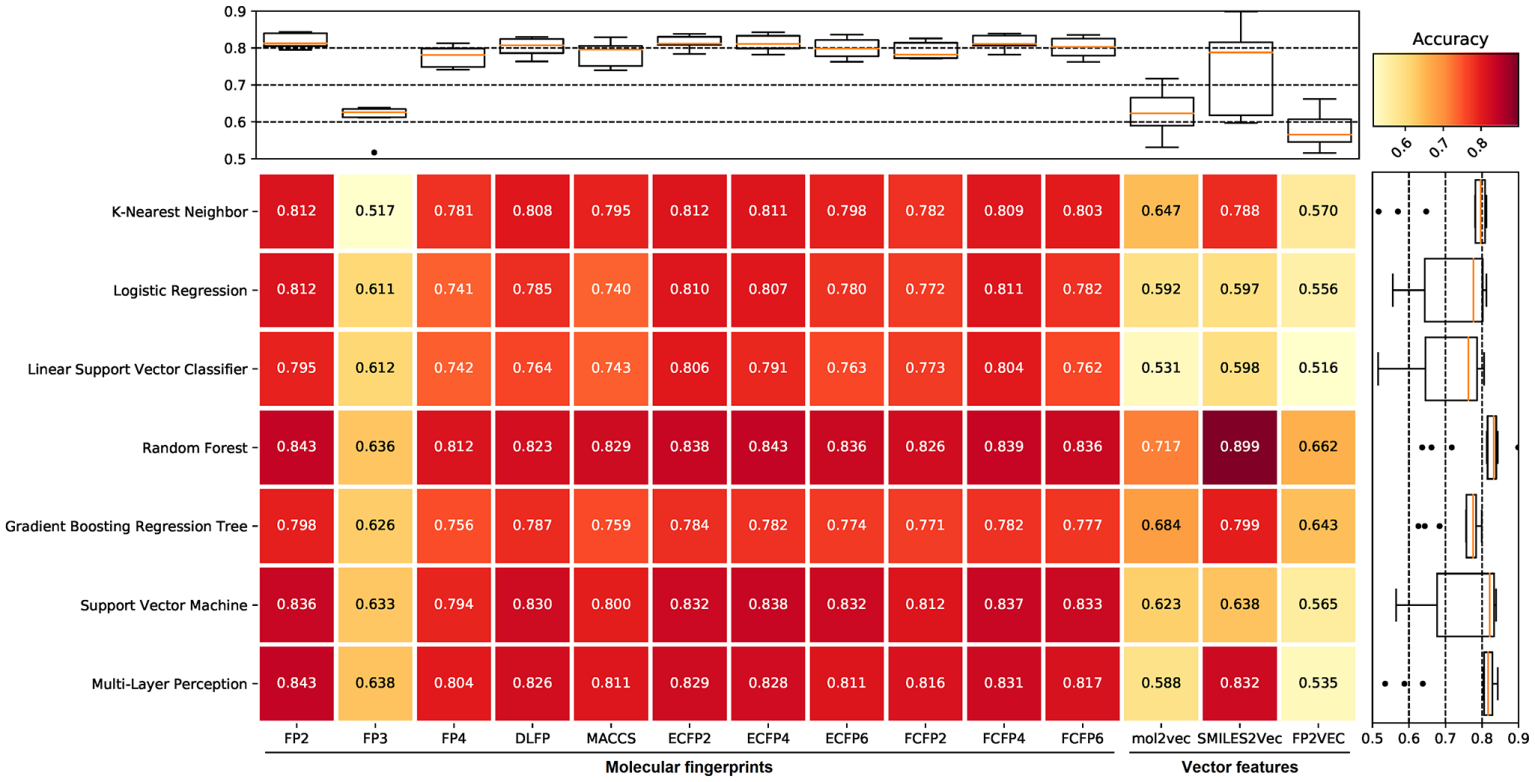
**The prediction accuracy of different machine learning methods for benchmark datasets.** The filtered datasets include one positive dataset and 10 negative datasets, therefore, each value in the figure is the average of 10 prediction accuracy. Compared with other machine learning methods, random forest (RF), support vector machine (SVM), and multi-layer perception (MLP) all show higher prediction accuracy. The benchmark dataset based on FP2 molecular fingerprints shows the highest prediction accuracy in the RF and MLP methods, and also shows high prediction accuracy in the SVM method among all molecular fingerprints. The accuracy fluctuates greatly among different machine learning methods in the benchmark dataset based on vector features.

To choose the appropriate machine learning methods to construct the anti-bacterial compound prediction model, we evaluate the predictive performance of different machine learning methods including k-nearest neighbor (KNN), logistic regression (LR), linear support vector classifier (LSVC), random forest (RF), gradient boosting regression tree (GBRT), support vector machine (SVM), and multi-layer perception (MLP). In the initial screening process, each machine learning method used benchmark datasets constructed from different molecular fingerprints for training and prediction with default parameters. The benchmark dataset was split into the training set (accounting for 80%) and the validation set (accounting for 20%), and then performed a 5-fold cross-validation test. The results suggested that the benchmark dataset based on FP2 molecular fingerprints, along with the SVM, RF, and MLP methods showed excellent prediction accuracy among all machine learning methods and molecular fingerprints combinations, whereas the accuracy fluctuates greatly among different machine learning methods in the benchmark dataset based on vector features (Figure 1). Therefore, the benchmark dataset based on FP2 molecular fingerprints, and the RF, SVM, and MLP methods were selected in the subsequent analysis.

### The development process of antibacterial compound predictor

**Figure 2:**
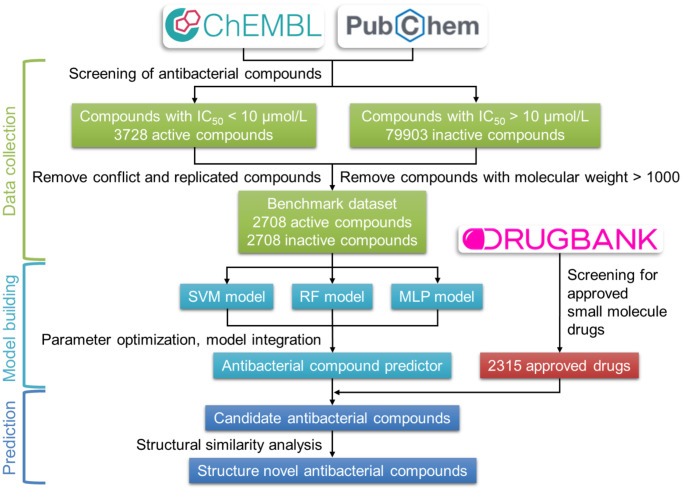
**Flow chart of the construction of the antibacterial compound prediction model.** The benchmark dataset was built using the active and inactive antibacterial compounds downloaded from the ChEMBL and the PubChem database. The combination of SVM, RF, and MLP methods was used to construct the antibacterial compounds predictor, which is used to predict the antibacterial activity of approved small-molecule drugs from the DrugBank database.

The development process of the antibacterial compound predictor is shown in Figure 2. The first step of the antibacterial compound predictor is to prepare the benchmark dataset using screened active and inactive antibacterial compounds from the ChEMBL and the PubChem database. Then, the SVM, RF, and MLP methods were used to build models using the benchmark dataset. Using the parameter grid search and 5-fold cross-validation strategy, the optimal parameters of these three models were determined (Table 1, Supplementary Figures 1–3). After training, parameter optimization, and model evaluation, the optimal SVM, RF, and MLP models were established. The final antibacterial compound predictor includes the combination of the optimal three models. The integrated model was used to predict the antibacterial activity of FDA-approved small-molecule drugs from the DrugBank database.

**Table 1:** Optimal parameters and prediction performance of different machine learning methods.

	**Support vector machine**	**Random forest**	**Multi-layer perception**
Optimal parameters	gamma: 0.01 C:10	n_estimators: 750	hidden_layer_sizes: 512 alpha: 0.0001
Accuracy	0.852 ± 0.002	0.849 ± 0.004	0.847 ± 0.004
Precision	0.854 ± 0.004	0.868 ± 0.004	0.850 ± 0.007
Sensitivity	0.850 ± 0.004	0.822 ± 0.007	0.845 ± 0.003
Specificity	0.854 ± 0.005	0.875 ± 0.004	0.850 ± 0.009
F1 score	0.852 ± 0.002	0.844 ± 0.005	0.847 ± 0.003
AUC	0.926 ± 0.002	0.932 ± 0.002	0.920 ± 0.002
MSE	0.148 ± 0.002	0.151 ± 0.004	0.153 ± 0.004

Abbreviations: AUC: area under the curve; MSE: mean squared error. Parameters of predictive performance were displayed as mean ± standard deviation.

### High performance of the SVM, RF, and MLP models

The overall performance of the SVM, RF, and MLP models was quantified by multiple classification evaluation indicators including accuracy, precision, sensitivity, specificity, F1 score, AUC, and MSE (Table 1). The mean values of accuracy, precision, sensitivity, specificity, and F1 score of the three models at around 0.85. The mean values of the AUC of these models were higher than 0.92 (ROC curves of these models shown in Supplementary Figure 4). The mean squared error of the three models is around 0.15. These indicate that all three models showed high effectiveness in antibacterial compounds prediction. Furthermore, the data showed that the standard deviations of these indicators are very small, suggesting that different negative datasets do not affect the overall performance of these models.

### Prediction of candidate antibacterial small-molecule drugs

**Figure 3:**
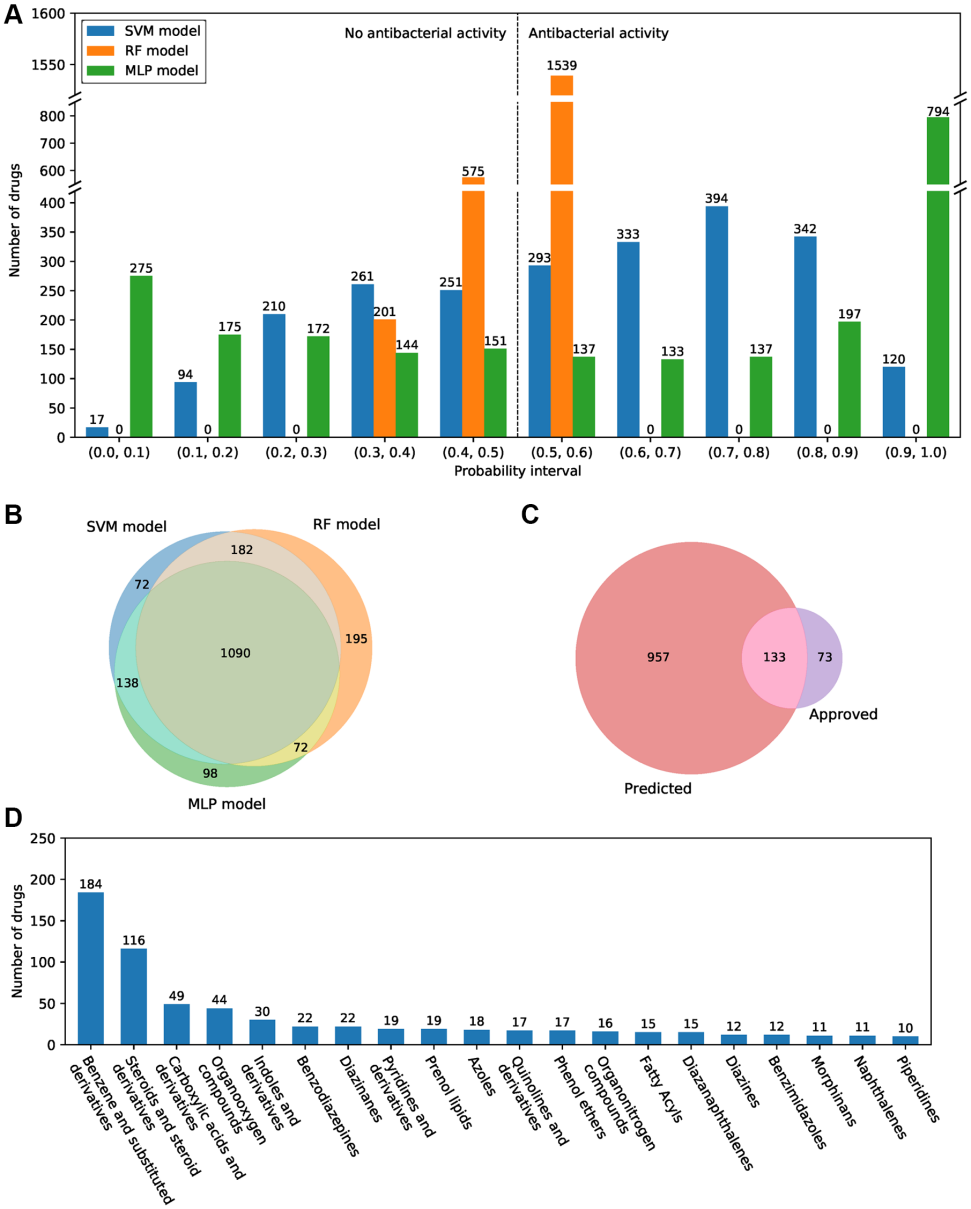
**Antibacterial prediction results of approved small-molecule drugs.** (**A**) The probability of predicted antibacterial activity for all small-molecule drugs in the SVM, RF, and MLP models. A drug with a probability value greater than 0.5 is considered an active antibacterial compound. (**B**) Venn diagram of the predicted antibacterial drugs in three machine learning models. (**C**) Venn diagram of the predicted antibacterial drugs and FDA-approved antibacterial drugs. (**D**) The top 20 categories of the 957 predicted novel antibacterial drugs.

All approved small-molecule drugs in the DrugBank database were used to screen for potential antibacterial compounds through the antibacterial compound predictor. The results showed that there are large differences in the number of drugs in isolated prediction intervals among the SVM, RF, and MLP models. There are more compounds in the probability intervals at both ends and fewer compounds in the middle intervals in the MLP model, whereas the distribution of predicted probabilities showed the opposite trend in the RF and SVM models (Figure 3A). There were 1482, 1539, and 1398 predicted active antibacterial drugs in the single SVM, RF, and MLP models. A total of 1090 drugs showed antibacterial activity shared by all three models (Figure 3B). The single model and the combination of the two models predicted relatively more active antibacterial compounds, and there is more overlap with FDA-approved drugs (Supplementary Figure 5). Among the prediction results by the combination of the three models, 133 antibacterial drugs were FDA approved (Figure 3C). Our results suggested that both the single models and the combination of multiple models all showed excellent prediction performance (Supplementary Table 1). Furthermore, for the remaining 957 drugs, many of them belong to benzene and substituted derivatives (184 drugs) and steroids and steroid derivatives (116 drugs), few drugs belong to other categories (Figure 3D).

### Structural similarity of the predicted antibacterial drugs

**Figure 4:**
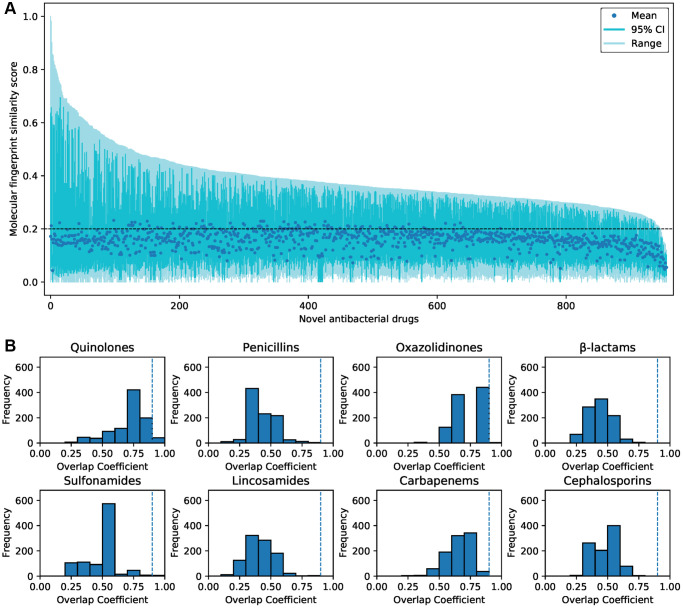
**The similarity of the predicted antibacterial drugs and FDA-approved antibacterial drugs.** (**A**) The molecular fingerprint similarity of 957 predicted novel antibacterial drugs and 206 FDA-approved antibacterial drugs. The average similarities between most of the predicted drugs and approved drugs were less than 0.2. (**B**) Substructure similarity between novel predicted antibacterial drugs and core scaffolds of approved antibacterial drugs. Compounds with an overlap coefficient higher than 0.9 are considered to have high substructure similarity.

Molecular fingerprint similarity was calculated between the predicted and FDA-approved antibacterial drugs. The predicted antibacterial drugs that are not approved for marketing were defined as novel predicted antibacterial drugs. There were low overall similarities between approved antibacterial drugs and novel predicted antibacterial drugs (Supplementary Figure 6). 873 novel-predicted antibacterial drugs showed average similarities ≤0.2 to all approved antibacterial drugs (Figure 4A). According to previous reports [32], we identified 8 representative core scaffolds from the FDA-approved antibacterial drugs (Supplementary Table 2). 906 predicted compounds do not contain any core scaffold (Figure 4B). Only 51 (5.3%) of the predicted compounds showed a high overlap coefficient with core scaffolds (Supplementary Table 3). These indicate that most of the predicted antibacterial drugs are structurally novel.

### Novel predicted antibacterial drugs

There were 9 novel-predicted drugs with an average similarity less than 0.1 and a maximum similarity less than 0.2 to all approved antibacterial drugs, and these drugs all showed high predicted probability in SVM, RF, and MLP models (Table 2). Details of these 9 drugs are listed in Supplementary Table 4. Among these drugs, cyclophosphamide (DB00531) and ifosfamide (DB01181) are anticancer drugs that were used to treat a variety of hematological tumors and solid tumors. Apraclonidine (DB00964) is used to relieve postsurgical ocular hypertension. Echothiophate is used for the treatment of subacute or chronic angle-closure glaucoma. The other 5 drugs are mainly used in general anesthesia, such as enflurane (DB00228), isoflurane (DB00753), methoxyflurane (DB01028), desflurane (DB01189), and sevoflurane (DB01236). To explore the correlation between these drugs and aging, 307 human aging-related genes were downloaded from the Human Ageing Genomic Resources (HAGR, https://genomics.senescence.info/). We used SEA [33], HitPickV2 [34], and TargetNet [35] for target prediction of these 9 drugs, the union set of the three predictions were chosen as target genes for the query drug. The results showed that these drugs may target recognized aging genes (such as APP, AR, RELA, and SIRT1, Supplementary Figure 7).

**Table 2:** The prediction results of 9 antibacterial drugs with low structural similarities.

**DrugBank ID**	**Name**	**Predicted probability**			**Structural similarity (mean (min-max))^1^**
		**SVM**	**RF**	**MLP**	
DB00228	Enflurane	0.741	0.544	0.916	0.055 (0.000–0.119)
DB00531	Cyclophosphamide	0.571	0.518	0.902	0.086 (0.010–0.150)
DB00753	Isoflurane	0.698	0.536	0.980	0.055 (0.000–0.120)
DB00964	Apraclonidine	0.514	0.514	0.501	0.093 (0.013–0.198)
DB01028	Methoxyflurane	0.770	0.504	0.913	0.048 (0.000–0.143)
DB01057	Echothiophate	0.703	0.518	0.864	0.072 (0.017–0.143)
DB01181	Ifosfamide	0.589	0.515	0.888	0.095 (0.010–0.172)
DB01189	Desflurane	0.732	0.546	0.975	0.055 (0.000–0.150)
DB01236	Sevoflurane	0.538	0.517	0.934	0.060 (0.000–0.162)

Abbreviations: SVM: support vector machine; RF: random forest; MLP: multi-layer perception. ^1^The structural similarities were calculated between the novel predicted antibacterial drugs and FDA-approved antibacterial drugs.

## DISCUSSION

Exploring the antibacterial activity of the approved drugs may be an effective way of screening new antibiotics. It is an effective approach by using machine learning methods to predict active antibacterial compounds [26, 28, 36]. The accuracy of the prediction model is affected by many factors, such as the quality of the benchmark datasets [37], the representative molecular characteristics of the compounds [16], the applicable machine learning models [9], and the optimized model parameters [38]. This study collected a large amount of experimental data on the antibacterial activity of compounds from the ChEMBL and PubChem databases. By comparing the prediction accuracy of multiple machine learning models on benchmark datasets constructed based on different molecular fingerprints, our results showed that the average prediction accuracy of SVM, RF, and MLP models are higher than other machine learning methods, and the FP2 molecular fingerprint is more representative than other fingerprints. Therefore, it is reasonable to construct the antibacterial compound predictor by building the benchmark datasets by calculating the FP2 molecular fingerprint of the compounds and combining the RF, SVM, and MLP models. However, the model constructed in this study did not achieve the desired prediction performance (only 133 of the 206 FDA-approved antibacterial drugs have been successfully predicted). This is probably because the benchmark datasets collected data from multiple sources and require a more effective data integration strategy. Furthermore, it is worth noting that parameter optimization can only slightly improve (approximately 1%) the prediction accuracy of the different machine learning models.

Through structural similarity analysis of the predicted active antibacterial drugs, we screened 9 drugs with novel structures. Apraclonidine is mainly used for the prevention and treatment of post-surgical intraocular pressure (IOP) elevation, and it is also indicated for the short-term adjunctive treatment of glaucoma [39]. Echothiophate is used in the treatment of subacute or chronic angle-closure glaucoma and some cases it is also used as accommodative esotropia [40]. Cyclophosphamide and ifosfamide are widely used broad-spectrum anticancer drugs [41, 42]. Studies showed that cyclophosphamide can inhibit bacterial translocation of the gastrointestinal tract [43] and reduce the abundance of lactobacilli and enterococci [44] in mice. Desflurane, enflurane, isoflurane, methoxyflurane, and sevoflurane are widely used volatile anesthetics [45, 46], most of these anesthetics have demonstrated antibacterial properties *in vitro* [47–50]. An early *in vitro* experiment showed that methoxyflurane and isoflurane exhibited excellent antibacterial activity, while enflurane had less effect on a few pathogens [48]. The resistance experiment to a variety of bacteria showed that isoflurane has higher antibacterial activity than sevoflurane [49]. Based on these reports, the antibacterial compound screening method used in this study is credible.

There are still many difficulties in the discovery of antibacterial compounds *in silico*. Firstly, the prediction accuracy is affected by the size and quality of the benchmark dataset. The definition of the active or inactive antibacterial compounds in this study is based on the *in vitro* experimental data. However, most of the screened active antibacterial compounds have not yet entered clinical trials, the human safety and clinical effectiveness of these compounds are still unclear [51]. Then, the compounds in this study were characterized by molecular fingerprints, whereas this method cannot reflect the complete structural features of given compounds and is not suitable for macromolecular compounds [16]. Next, machine learning models need further optimization. The prediction accuracy of the SVM, RF, and MLP models in this study is around 0.85, optimizing these models may be able to obtain higher prediction accuracy. Lastly, considering that compounds may produce different types of molecules during the metabolic process, computational simulation of the drug metabolic process [52] in humans will make the predictions more convincing.

The development of new and highly effective antibiotics can alleviate the crisis of bacterial infections, extend human lifespan, and reduce the incidence of infectious diseases in the elderly. This study provides a new insight for predicting antibacterial compounds with novel structures by using approved drugs. The existing approach could be extended by different augmentation methods (such as compound augmentation by graph or molecular description) with different machine learning state-of-the-art methods such as deep-learning methods. There are still many challenges and opportunities in using machine learning to predict antibacterial compounds. With the development of big data technology, the continuous optimization of machine learning models and algorithms, and the discovery of more antibacterial active compounds and drugs, it is foreseeable that the prediction of antibacterial compounds in the future will achieve higher accuracy and credibility.

## MATERIALS AND METHODS

### Antibacterial compounds collection

Compounds that performed antimicrobial activity tests were collected from ChEMBLdb (version 25, https://www.ebi.ac.uk/chembl/) and PubChem (https://pubchem.ncbi.nlm.nih.gov/) databases. A total of 83768 compounds were obtained, 8001 of these compounds have a clear IC50 value, and others only have an inactive label. The IC50 cutoff value of antibacterial activity was defined by curve fitting the IC50 values of all compounds. Compounds with IC50 less than 10 μmol/L were generally considered as active antibacterial compounds [53–57], the curve fitting results also suggest that this cutoff is reasonable (Supplementary Figure 8). Based on the curve fitting results, compounds with IC50 higher than 10 μmol/L were considered inactive antibacterial compounds. Pybel, a python wrapper of OpenBabel [58, 59] was used to access the SMILES string of compounds and calculate molecular fingerprint which represents the presence or absence of particular substructures in the molecule. Multiple types of molecular fingerprints of all compounds were calculated. Benchmark datasets were built based on the following steps: (1) remove duplicate compounds; (2) remove compounds with a molecular weight greater than 1000; (3) remove compounds with molecular fingerprint similarities higher than 0.9 between the active and inactive antibacterial compounds. Finally, we got a positive dataset including 2708 active antibacterial compounds and a negative dataset including 78620 inactive antibacterial compounds. All active antibacterial compounds have IC50 values whereas only 1893 inactive antibacterial compounds have IC50 values.

### Construction of the benchmark dataset

There is a large difference in the number of compounds between the positive and negative datasets. The positive dataset contains 2708 active antibacterial compounds. To balance the number of compounds between the positive and negative datasets, the filtered negative dataset contains 1893 inactive antibacterial compounds with IC50 values, the remaining quantity difference was randomly selected from the inactive antibacterial compounds only with an inactive label. Considering the uncertainty of random selection, we repeated 10 times for negative dataset extract. Therefore, the filtered datasets including one positive dataset and 10 negative datasets, each negative dataset are combined with the positive data set for subsequent analysis. Next, the molecular fingerprint is calculated for the positive dataset and all repeated negative datasets. The following types of molecular fingerprints were calculated including FP2, FP3, FP4, DLFP, MACCS, ECFP2, ECFP4, ECFP6, FCFP2, FCFP4, and FCFP6. Several start-of-the-art chemoinformatics approaches were also calculated such as mol2vec [60], SMILES2Vec [61], and FP2VEC [62]. The features of each compound were presented by the binary bits of the different types of molecular fingerprints or vectors and these features were used for machine learning modeling (Supplementary Table 5). All these benchmark datasets were used for the preliminary screening of applicable machine learning models.

### Parameter selection of the SVM, RF, and MLP models

The SVM, RF, and MLP models for antibacterial compounds prediction were built using the svm, ensemble, and neural_network module in the scikit-learn Python library (version: 0.20.0, https://scikit-learn.org/stable/). A parameter grid search strategy was used to choose the optimal parameter "gamma" for the kernel function and regularization parameter "C" for the SVM model, the optimal number of trees (parameter "n_estimators") for the RF model, and the optimal hidden layer sizes and alpha for the MLP model. The other parameters of the above three models use default values. The benchmark dataset was randomly split into the training and validation set (accounting for 80%) and the test set (accounting for 20%) using the train_test_split function in the scikit-learn. The 5-fold cross-validation method was used to evaluate the generalization performance of the model with specified parameters in the training and validation set. The cross-validation accuracy was calculated for model evaluation. After cross-validation, a temporary model was built using the training and validation set and calculated the area under the curve (AUC) for the receiver operating characteristic (ROC) curve in the test set. Considering that there may be similar compounds in the split datasets, dataset split and cross-validation were repeated 10 times, which may reduce the impact of similar compounds on the prediction performance of these models. For each given parameter, the mean cross-validation accuracy and mean AUC was calculated. The optimal model was selected by comparing the maximum mean cross-validation accuracy under different parameters. If there were multiple models with the same mean accuracy, the model with the maximum AUC was considered to be the optimal model.

### Performance evaluation

The optimal SVM, RF, and MLP models were used for performance evaluation. The confusion matrix was calculated using the results of the optimal cross-validation test. The true positive (TP) indicates the number of correctly predicted active antibacterial compounds, the true negative (TN) indicates the number of correctly predicted inactive antibacterial compounds, the false positive (FP) indicates the number of inactive antibacterial compounds predicted as active antibacterial compounds, and the false negative (FN) indicates the number of active antibacterial compounds predicted as inactive antibacterial compounds. We calculated the following quality indices: accuracy = (TP + TN)/(TP + TN + FP + FN), precision = TP/(TP + FP), sensitivity = TP/(TP + FN), specificity = TN/(TN + FP), and F1 score = 2 × TP/(2 × TP + FP + FN). Mean squared error (MSE) was calculated for all three models. Because the filtered datasets include one positive dataset and 10 negative datasets, the average of 10 calculations of these quality indices and AUC were used to evaluate the SVM, RF, and MLP model performance. A model with high scores (≥0.8) of accuracy, precision, F1 score, and AUC was considered to be an effective model.

### Antibacterial small-molecule drugs prediction

The final SVM, RF, and MLP models were built using the benchmark dataset with the optimal parameters. All these three models were used to predict antibacterial activity for approved small-molecule drugs. We compared the prediction performance of a single model and a combination of different models. The candidate antibacterial drugs were defined as the drugs that showed antibacterial activity in all the SVM, RF, and MLP models. Drug information was acquired from the DrugBank database (https://www.drugbank.ca/) [63]. We first filtered the drugs with approved status but not withdrawn yet, then removed the drugs with molecular weight ≥1000. Finally, 2315 approved small-molecule drugs were screened to perform antibacterial activity prediction. The predicted active antibacterial drugs excluding FDA-approved antibacterial drugs were defined as novel antibacterial drugs.

### Structural similarity analysis

FP2 molecular fingerprint similarity was calculated among all novel antibacterial drugs and FDA-approved antibacterial drugs. The overlap between fingerprints is quantified as a measure of molecular similarity using the Tanimoto coefficient (Tc). The predicted drugs with average and maximum molecular fingerprint similarity less than 0.1 and 0.2 were considered to be structurally novel. Furthermore, previous literature reported several core scaffolds shared by most antibacterial compounds [32]. The flexible maximum common substructure algorithms in the fmcsR package [64] in R were used to identify whether the core scaffolds exist in the predicted antibacterial drugs.

## Supplementary Materials

Supplementary Figures

Supplementary Tables

## References

[1] Allen JC, Toapanta FR, Chen W, Tennant SM. Understanding immunosenescence and its impact on vaccination of older adults. Vaccine. 2020; 38:8264–72. 10.1016/j.vaccine.2020.11.002 33229108 PMC7719605

[2] Brown GC. The endotoxin hypothesis of neurodegeneration. J Neuroinflammation. 2019; 16:180. 10.1186/s12974-019-1564-7 31519175 PMC6744684

[3] Panza F, Lozupone M, Solfrizzi V, Watling M, Imbimbo BP. Time to test antibacterial therapy in Alzheimer's disease. Brain. 2019; 142:2905–29. 10.1093/brain/awz244 31532495

[4] Tacconelli E, Carrara E, Savoldi A, Harbarth S, Mendelson M, Monnet DL, Pulcini C, Kahlmeter G, Kluytmans J, Carmeli Y, Ouellette M, Outterson K, Patel J, et al, and WHO Pathogens Priority List Working Group. Discovery, research, and development of new antibiotics: the WHO priority list of antibiotic-resistant bacteria and tuberculosis. Lancet Infect Dis. 2018; 18:318–27. 10.1016/S1473-3099(17)30753-3 29276051

[5] Jackson N, Czaplewski L, Piddock LJV. Discovery and development of new antibacterial drugs: learning from experience? J Antimicrob Chemother. 2018; 73:1452–9. 10.1093/jac/dky019 29438542

[6] Pereira DA, Williams JA. Origin and evolution of high throughput screening. Br J Pharmacol. 2007; 152:53–61. 10.1038/sj.bjp.0707373 17603542 PMC1978279

[7] Mishra KP, Ganju L, Sairam M, Banerjee PK, Sawhney RC. A review of high throughput technology for the screening of natural products. Biomed Pharmacother. 2008; 62:94–8. 10.1016/j.biopha.2007.06.012 17692498

[8] Tommasi R, Brown DG, Walkup GK, Manchester JI, Miller AA. ESKAPEing the labyrinth of antibacterial discovery. Nat Rev Drug Discov. 2015; 14:529–42. 10.1038/nrd4572 26139286

[9] Durrant JD, Amaro RE. Machine-learning techniques applied to antibacterial drug discovery. Chem Biol Drug Des. 2015; 85:14–21. 10.1111/cbdd.12423 25521642 PMC4273861

[10] Nicolaou CA, Brown N. Multi-objective optimization methods in drug design. Drug Discov Today Technol. 2013; 10:e427–35. 10.1016/j.ddtec.2013.02.001 24050140

[11] Cummins DJ, Bell MA. Integrating Everything: The Molecule Selection Toolkit, a System for Compound Prioritization in Drug Discovery. J Med Chem. 2016; 59:6999–7010. 10.1021/acs.jmedchem.5b01338 26950497

[12] Halder AK, Dias Soeiro Cordeiro MN. QSAR-Co-X: an open source toolkit for multitarget QSAR modelling. J Cheminform. 2021; 13:29. 10.1186/s13321-021-00508-0 33858509 PMC8048082

[13] Sánchez-Rodríguez A, Pérez-Castillo Y, Schürer SC, Nicolotti O, Mangiatordi GF, Borges F, Cordeiro MND, Tejera E, Medina-Franco JL, Cruz-Monteagudo M. From flamingo dance to (desirable) drug discovery: a nature-inspired approach. Drug Discov Today. 2017; 22:1489–502. 10.1016/j.drudis.2017.05.008 28624633 PMC5650527

[14] Speck-Planche A, Cordeiro MN. Simultaneous modeling of antimycobacterial activities and ADMET profiles: a chemoinformatic approach to medicinal chemistry. Curr Top Med Chem. 2013; 13:1656–65. 10.2174/15680266113139990116 23889052

[15] Cereto-Massagué A, Ojeda MJ, Valls C, Mulero M, Garcia-Vallvé S, Pujadas G. Molecular fingerprint similarity search in virtual screening. Methods. 2015; 71:58–63. 10.1016/j.ymeth.2014.08.005 25132639

[16] Muegge I, Mukherjee P. An overview of molecular fingerprint similarity search in virtual screening. Expert Opin Drug Discov. 2016; 11:137–48. 10.1517/17460441.2016.1117070 26558489

[17] Vamathevan J, Clark D, Czodrowski P, Dunham I, Ferran E, Lee G, Li B, Madabhushi A, Shah P, Spitzer M, Zhao S. Applications of machine learning in drug discovery and development. Nat Rev Drug Discov. 2019; 18:463–77. 10.1038/s41573-019-0024-5 30976107 PMC6552674

[18] Chen X, Yan CC, Zhang X, Zhang X, Dai F, Yin J, Zhang Y. Drug-target interaction prediction: databases, web servers and computational models. Brief Bioinform. 2016; 17:696–712. 10.1093/bib/bbv066 26283676

[19] D'Souza S, Prema KV, Balaji S. Machine learning models for drug-target interactions: current knowledge and future directions. Drug Discov Today. 2020; 25:748–56. 10.1016/j.drudis.2020.03.003 32171918

[20] Walters WP, Barzilay R. Applications of Deep Learning in Molecule Generation and Molecular Property Prediction. Acc Chem Res. 2021; 54:263–70. 10.1021/acs.accounts.0c00699 33370107

[21] Ekins S, Puhl AC, Zorn KM, Lane TR, Russo DP, Klein JJ, Hickey AJ, Clark AM. Exploiting machine learning for end-to-end drug discovery and development. Nat Mater. 2019; 18:435–41. 10.1038/s41563-019-0338-z 31000803 PMC6594828

[22] Lata S, Sharma BK, Raghava GP. Analysis and prediction of antibacterial peptides. BMC Bioinformatics. 2007; 8:263. 10.1186/1471-2105-8-263 17645800 PMC2041956

[23] Khosravian M, Faramarzi FK, Beigi MM, Behbahani M, Mohabatkar H. Predicting antibacterial peptides by the concept of Chou's pseudo-amino acid composition and machine learning methods. Protein Pept Lett. 2013; 20:180–6. 10.2174/092986613804725307 22894156

[24] Zoffmann S, Vercruysse M, Benmansour F, Maunz A, Wolf L, Blum Marti R, Heckel T, Ding H, Truong HH, Prummer M, Schmucki R, Mason CS, Bradley K, et al. Machine learning-powered antibiotics phenotypic drug discovery. Sci Rep. 2019; 9:5013. 10.1038/s41598-019-39387-9 30899034 PMC6428806

[25] Rahman SF, Olm MR, Morowitz MJ, Banfield JF. Machine Learning Leveraging Genomes from Metagenomes Identifies Influential Antibiotic Resistance Genes in the Infant Gut Microbiome. mSystems. 2018; 3:e00123–17. 10.1128/mSystems.00123-17 29359195 PMC5758725

[26] Stokes JM, Yang K, Swanson K, Jin W, Cubillos-Ruiz A, Donghia NM, MacNair CR, French S, Carfrae LA, Bloom-Ackermann Z, Tran VM, Chiappino-Pepe A, Badran AH, et al. A Deep Learning Approach to Antibiotic Discovery. Cell. 2020; 180:688–702.e13. 10.1016/j.cell.2020.01.021 32084340 PMC8349178

[27] Yang XG, Chen D, Wang M, Xue Y, Chen YZ. Prediction of antibacterial compounds by machine learning approaches. J Comput Chem. 2009; 30:1202–11. 10.1002/jcc.21148 18988254

[28] Kavvas ES, Catoiu E, Mih N, Yurkovich JT, Seif Y, Dillon N, Heckmann D, Anand A, Yang L, Nizet V, Monk JM, Palsson BO. Machine learning and structural analysis of Mycobacterium tuberculosis pan-genome identifies genetic signatures of antibiotic resistance. Nat Commun. 2018; 9:4306. 10.1038/s41467-018-06634-y 30333483 PMC6193043

[29] Niehaus KE, Walker TM, Crook DW, Peto TEA, Clifton DA. Machine learning for the prediction of antibacterial susceptibility in Mycobacterium tuberculosis. IEEE-EMBS International Conference on Biomedical and Health Informatics (BHI). 2014; 618–21. 10.1109/BHI.2014.6864440

[30] Hefti FF. Requirements for a lead compound to become a clinical candidate. BMC Neurosci. 2008 (Suppl 3); 9:S7. 10.1186/1471-2202-9-S3-S7 19091004 PMC2604885

[31] Savoia D. New Antimicrobial Approaches: Reuse of Old Drugs. Curr Drug Targets. 2016; 17:731–8. 10.2174/1389450116666150806124110 26245476

[32] Fair RJ, Tor Y. Antibiotics and bacterial resistance in the 21st century. Perspect Medicin Chem. 2014; 6:25–64. 10.4137/PMC.S14459 25232278 PMC4159373

[33] Keiser MJ, Roth BL, Armbruster BN, Ernsberger P, Irwin JJ, Shoichet BK. Relating protein pharmacology by ligand chemistry. Nat Biotechnol. 2007; 25:197–206. 10.1038/nbt1284 17287757

[34] Hamad S, Adornetto G, Naveja JJ, Chavan Ravindranath A, Raffler J, Campillos M. HitPickV2: a web server to predict targets of chemical compounds. Bioinformatics. 2019; 35:1239–40. 10.1093/bioinformatics/bty759 30169615

[35] Yao ZJ, Dong J, Che YJ, Zhu MF, Wen M, Wang NN, Wang S, Lu AP, Cao DS. TargetNet: a web service for predicting potential drug-target interaction profiling via multi-target SAR models. J Comput Aided Mol Des. 2016; 30:413–24. 10.1007/s10822-016-9915-2 27167132

[36] Yang JH, Wright SN, Hamblin M, McCloskey D, Alcantar MA, Schrübbers L, Lopatkin AJ, Satish S, Nili A, Palsson BO, Walker GC, Collins JJ. A White-Box Machine Learning Approach for Revealing Antibiotic Mechanisms of Action. Cell. 2019; 177:1649–61.e9. 10.1016/j.cell.2019.04.016 31080069 PMC6545570

[37] Beltran JA, Aguilera-Mendoza L, Brizuela CA. Optimal selection of molecular descriptors for antimicrobial peptides classification: an evolutionary feature weighting approach. BMC Genomics. 2018; 19:672. 10.1186/s12864-018-5030-1 30255784 PMC6156846

[38] Díaz I. Machine learning in the estimation of causal effects: targeted minimum loss-based estimation and double/debiased machine learning. Biostatistics. 2020; 21:353–8. 10.1093/biostatistics/kxz042 31742333

[39] Harasymowycz P, Royer C, Cui AX, Barbeau M, Jobin-Gervais K, Mathurin K, Lachaine J, Beauchemin C. Short-term efficacy of latanoprostene bunod for the treatment of open-angle glaucoma and ocular hypertension: a systematic literature review and a network meta-analysis. Br J Ophthalmol. 2021. [Epub ahead of print]. 10.1136/bjophthalmol-2020-317262 33397657 PMC9046749

[40] Kini MM, Dahl AA, Roberts CR, Lehwalder LW, Grant WM. Echothiophate, pilocarpine, and open-angle glaucoma. Arch Ophthalmol. 1973; 89:190–2. 10.1001/archopht.1973.01000040192005 4691318

[41] Emadi A, Jones RJ, Brodsky RA. Cyclophosphamide and cancer: golden anniversary. Nat Rev Clin Oncol. 2009; 6:638–47. 10.1038/nrclinonc.2009.146 19786984

[42] Matz EL, Hsieh MH. Review of Advances in Uroprotective Agents for Cyclophosphamide- and Ifosfamide-induced Hemorrhagic Cystitis. Urology. 2017; 100:16–9. 10.1016/j.urology.2016.07.030 27566144

[43] Suzuki T, Itoh K, Hagiwara T, Nakayama H, Honjyo K, Hirota Y, Kaneko T, Suzuki H. Inhibition of bacterial translocation from the gastrointestinal tract of mice injected with cyclophosphamide. Curr Microbiol. 1996; 33:78–83. 10.1007/s002849900078 8662176

[44] Alexander JL, Wilson ID, Teare J, Marchesi JR, Nicholson JK, Kinross JM. Gut microbiota modulation of chemotherapy efficacy and toxicity. Nat Rev Gastroenterol Hepatol. 2017; 14:356–65. 10.1038/nrgastro.2017.20 28270698

[45] Kharasch ED, Thummel KE. Identification of cytochrome P450 2E1 as the predominant enzyme catalyzing human liver microsomal defluorination of sevoflurane, isoflurane, and methoxyflurane. Anesthesiology. 1993; 79:795–807. 10.1097/00000542-199310000-00023 8214760

[46] Terrell RC, Warner DS. The invention and development of enflurane, isoflurane, sevoflurane, and desflurane. Anesthesiology. 2008; 108:531–3. 10.1097/ALN.0b013e31816499cc 18292690

[47] Horton JN, Sussman M, Mushin WW. The antibacterial action of anaesthetic vapours. Br J Anaesth. 1970; 42:483–7. 10.1093/bja/42.6.483 4912643

[48] Giorgi A, Parodi F, Piacenza G, Mantellini E, Salio M, Cremonte LG, Grosso E. [Antibacterial and antifungal activity of isoflurane and common anesthetic gases]. Minerva Med. 1986; 77:2007–10. 3534634

[49] Martínez-Serrano M, Gerónimo-Pardo M, Martínez-Monsalve A, Crespo-Sánchez MD. Antibacterial effect of sevoflurane and isoflurane. Rev Esp Quimioter. 2017; 30:84–9. 28198170

[50] Imbernón-Moya A, Ortiz-de Frutos FJ, Sanjuan-Alvarez M, Portero-Sanchez I, Merinero-Palomares R, Alcazar V. Topical sevoflurane for chronic venous ulcers infected by multi-drug-resistant organisms. Int Wound J. 2017; 14:1388–90. 10.1111/iwj.12794 28736974 PMC7950177

[51] Pogodin PV, Lagunin AA, Rudik AV, Druzhilovskiy DS, Filimonov DA, Poroikov VV. AntiBac-Pred: A Web Application for Predicting Antibacterial Activity of Chemical Compounds. J Chem Inf Model. 2019; 59:4513–8. 10.1021/acs.jcim.9b00436 31661960

[52] Roy H, Nandi S. In-Silico Modeling in Drug Metabolism and Interaction: Current Strategies of Lead Discovery. Curr Pharm Des. 2019; 25:3292–305. 10.2174/1381612825666190903155935 31481001

[53] Choi KE, Balupuri A, Kang NS. The Study on the hERG Blocker Prediction Using Chemical Fingerprint Analysis. Molecules. 2020; 25:2615. 10.3390/molecules25112615 32512802 PMC7321128

[54] Lind AP, Anderson PC. Predicting drug activity against cancer cells by random forest models based on minimal genomic information and chemical properties. PLoS One. 2019; 14:e0219774. 10.1371/journal.pone.0219774 31295321 PMC6622537

[55] Deng YH, Wang NN, Zou ZX, Zhang L, Xu KP, Chen AF, Cao DS, Tan GS. Multi-Target Screening and Experimental Validation of Natural Products from *Selaginella* Plants against Alzheimer's Disease. Front Pharmacol. 2017; 8:539. 10.3389/fphar.2017.00539 28890698 PMC5574911

[56] Rifaioglu AS, Nalbat E, Atalay V, Martin MJ, Cetin-Atalay R, Doğan T. DEEPScreen: high performance drug-target interaction prediction with convolutional neural networks using 2-D structural compound representations. Chem Sci. 2020; 11:2531–57. 10.1039/c9sc03414e 33209251 PMC7643205

[57] Li GH, Huang JF. CDRUG: a web server for predicting anticancer activity of chemical compounds. Bioinformatics. 2012; 28:3334–5. 10.1093/bioinformatics/bts625 23080119

[58] O'Boyle NM, Morley C, Hutchison GR. Pybel: a Python wrapper for the OpenBabel cheminformatics toolkit. Chem Cent J. 2008; 2:5. 10.1186/1752-153x-2-5 18328109 PMC2270842

[59] O'Boyle NM, Banck M, James CA, Morley C, Vandermeersch T, Hutchison GR. Open Babel: An open chemical toolbox. J Cheminform. 2011; 3:33. 10.1186/1758-2946-3-33 21982300 PMC3198950

[60] Jaeger S, Fulle S, Turk S. Mol2vec: Unsupervised Machine Learning Approach with Chemical Intuition. J Chem Inf Model. 2018; 58:27–35. 10.1021/acs.jcim.7b00616 29268609

[61] Öztürk H, Ozkirimli E, Özgür A. A novel methodology on distributed representations of proteins using their interacting ligands. Bioinformatics. 2018; 34:i295–303. 10.1093/bioinformatics/bty287 29949957 PMC6022674

[62] Jeon W, Kim D. FP2VEC: a new molecular featurizer for learning molecular properties. Bioinformatics. 2019; 35:4979–85. 10.1093/bioinformatics/btz307 31070725

[63] Wishart DS, Knox C, Guo AC, Shrivastava S, Hassanali M, Stothard P, Chang Z, Woolsey J. DrugBank: a comprehensive resource for in silico drug discovery and exploration. Nucleic Acids Res. 2006; 34:D668–72. 10.1093/nar/gkj067 16381955 PMC1347430

[64] Wang Y, Backman TW, Horan K, Girke T. fmcsR: mismatch tolerant maximum common substructure searching in R. Bioinformatics. 2013; 29:2792–4. 10.1093/bioinformatics/btt475 23962615

