# Supplementary material for: Screening of antibacterial compounds with novel structure from the FDA approved drugs using machine learning methods
Source: Aging (Albany NY). 2022 Feb 12;14(3):1448–72. doi: 10.18632/aging.203887 (PMC8876917; doi:10.18632/aging.203887)
Supplement: Supplementary Figures [file aging-14-203887-s001.pdf]

SUPPLEMENTARY FIGURES

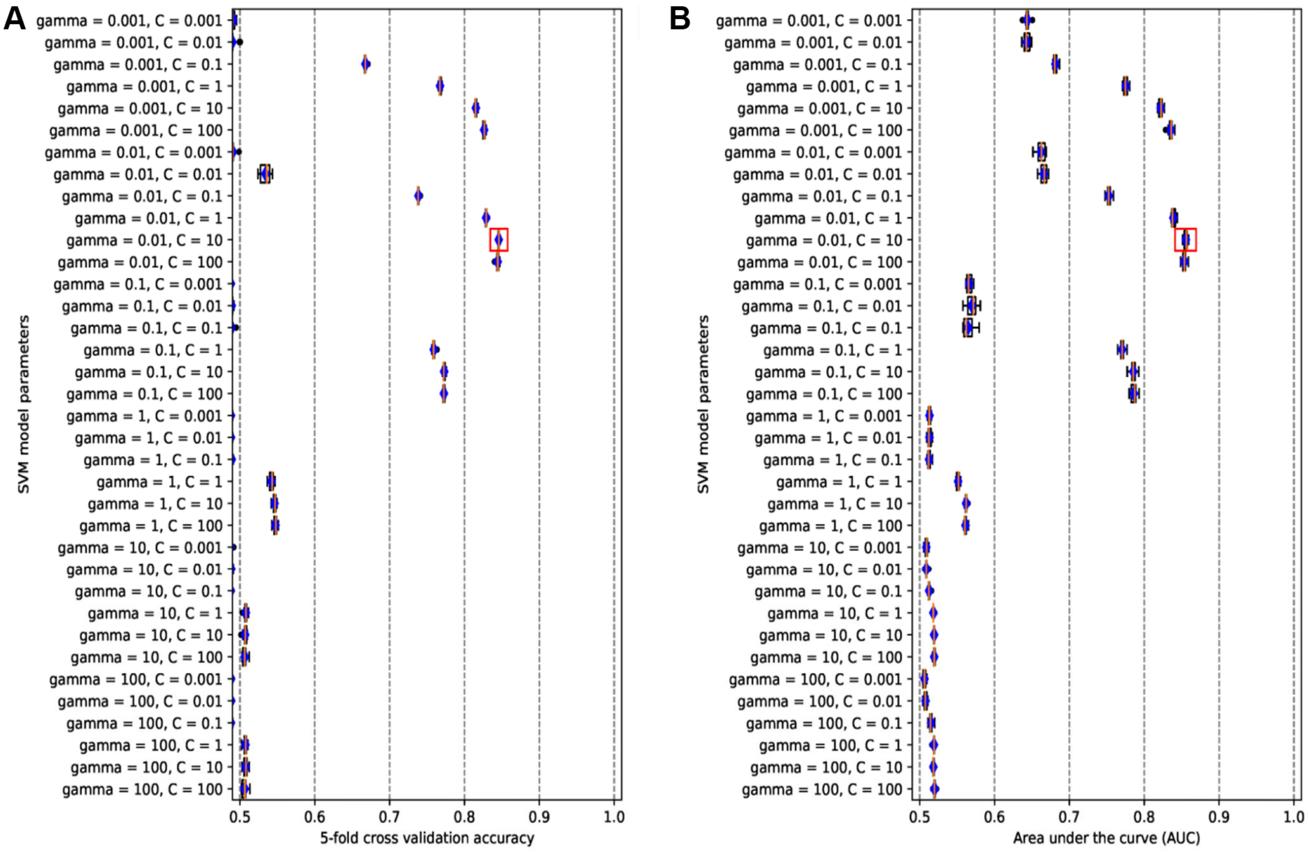

Supplementary Figure 1. Parameter optimization of the support vector machine (SVM) model. The optimal parameter "gamma" and "C" was determined according to the maximum 5-fold cross-validation accuracy (**A**) and the maximum area under the curve (AUC) (**B**). The red box indicates the maximum 5-fold cross-validation accuracy or AUC.

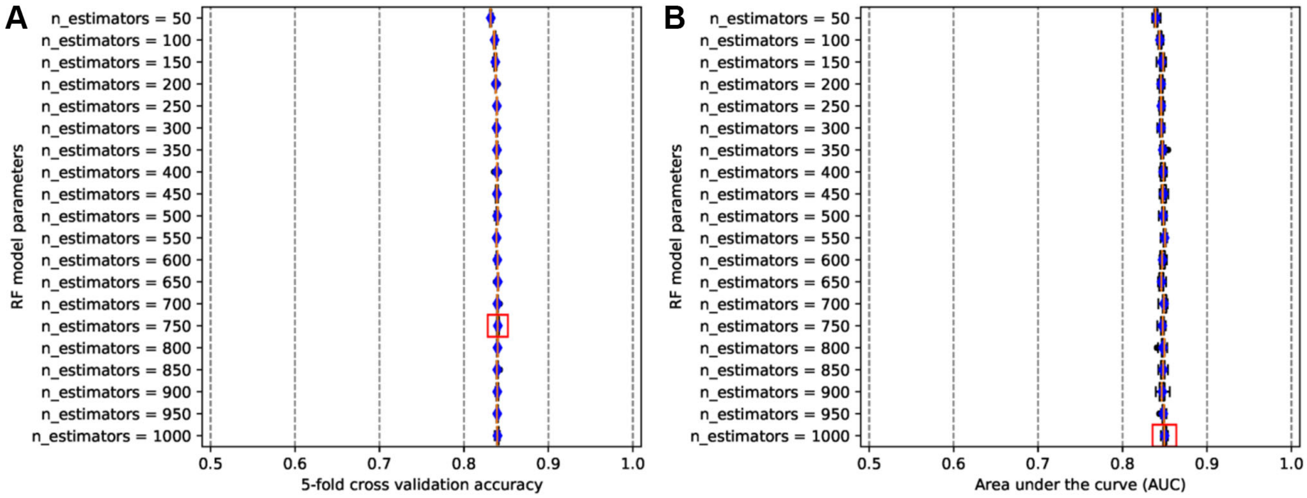

Supplementary Figure 2. Parameter optimization of the random forest (RF) model. The optimal parameter "n_estimators" was determined according to the maximum 5-fold cross-validation accuracy (**A**) and the maximum area under the curve (AUC) (**B**). The red box indicates the maximum 5-fold cross-validation accuracy or AUC.

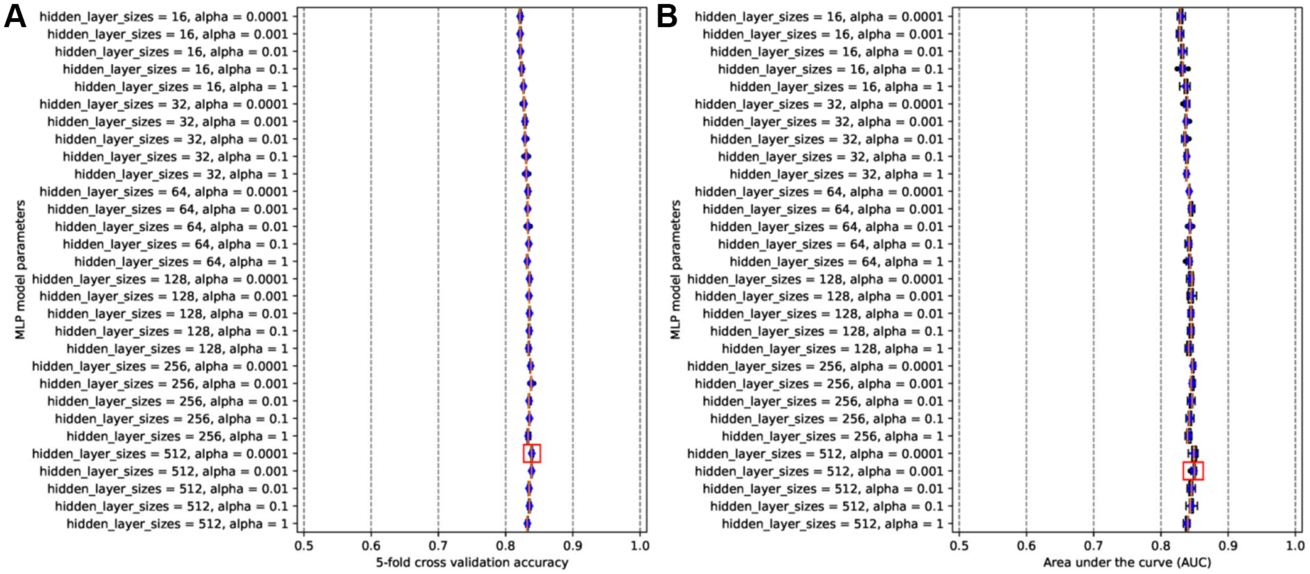

Supplementary Figure 3. Parameter optimization of the multi-layer perception (MLP) model. The optimal parameters “hidden_layer_sizes” and “alpha” were determined according to the maximum 5-fold cross-validation accuracy (**A**) and the maximum area under the curve (AUC) (**B**). The red box indicates the maximum 5-fold cross-validation accuracy or AUC.

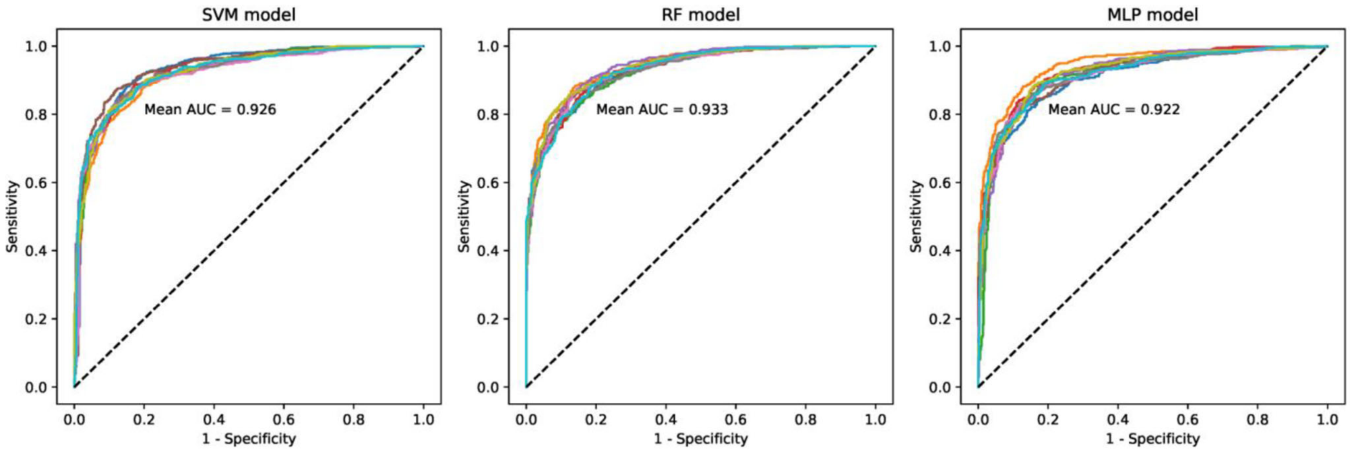

Supplementary Figure 4. The receiver operating characteristic (ROC) curve and area under the curve (AUC) for the optimal SVM, RF, and MLP models. ROC and AUC were calculated for each cross-validation, all 10 times were shown in the figure. The figure showed the use of one of the benchmark datasets (see Methods 2.2 and 2.5) for model construction and its prediction performance.

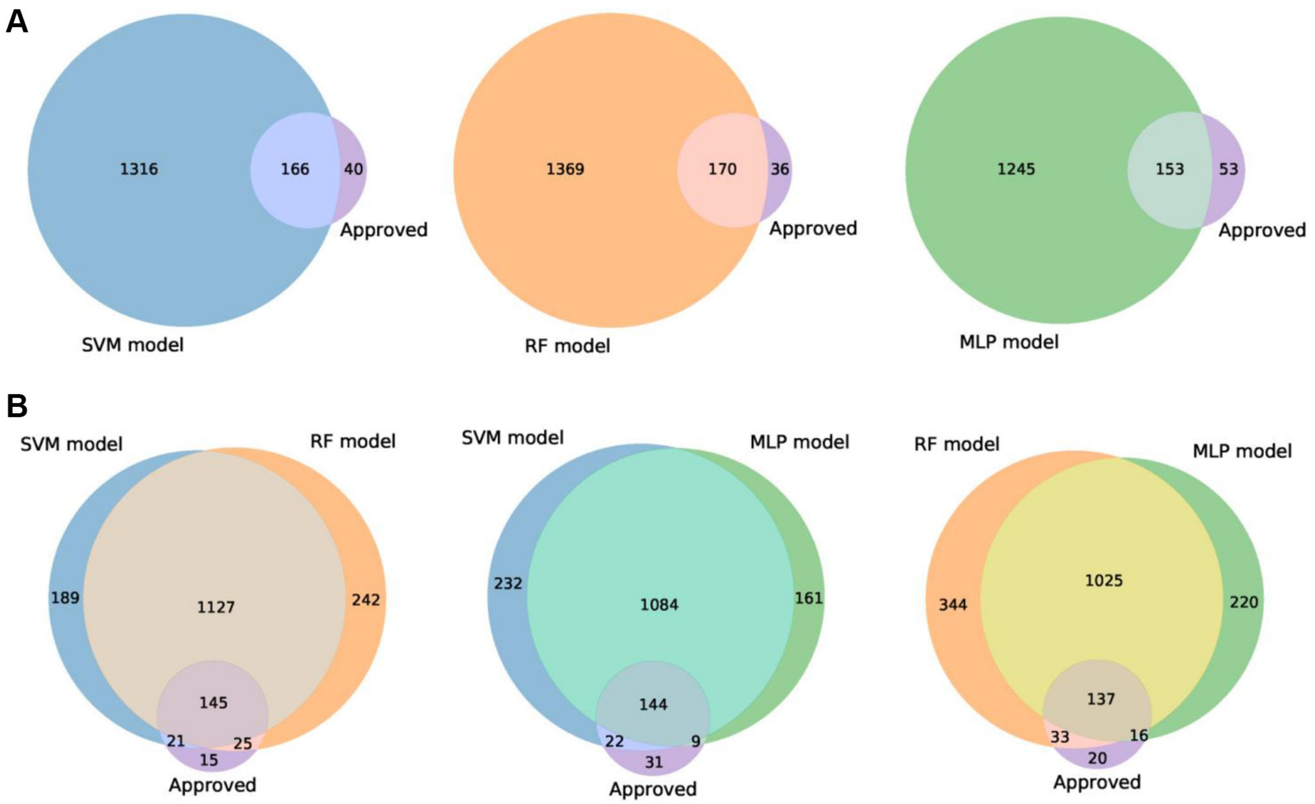

Supplementary Figure 5. Venn diagram of predicted and FDA-approved antibacterial drugs in a single model (**A**) and the combination of two models (**B**).

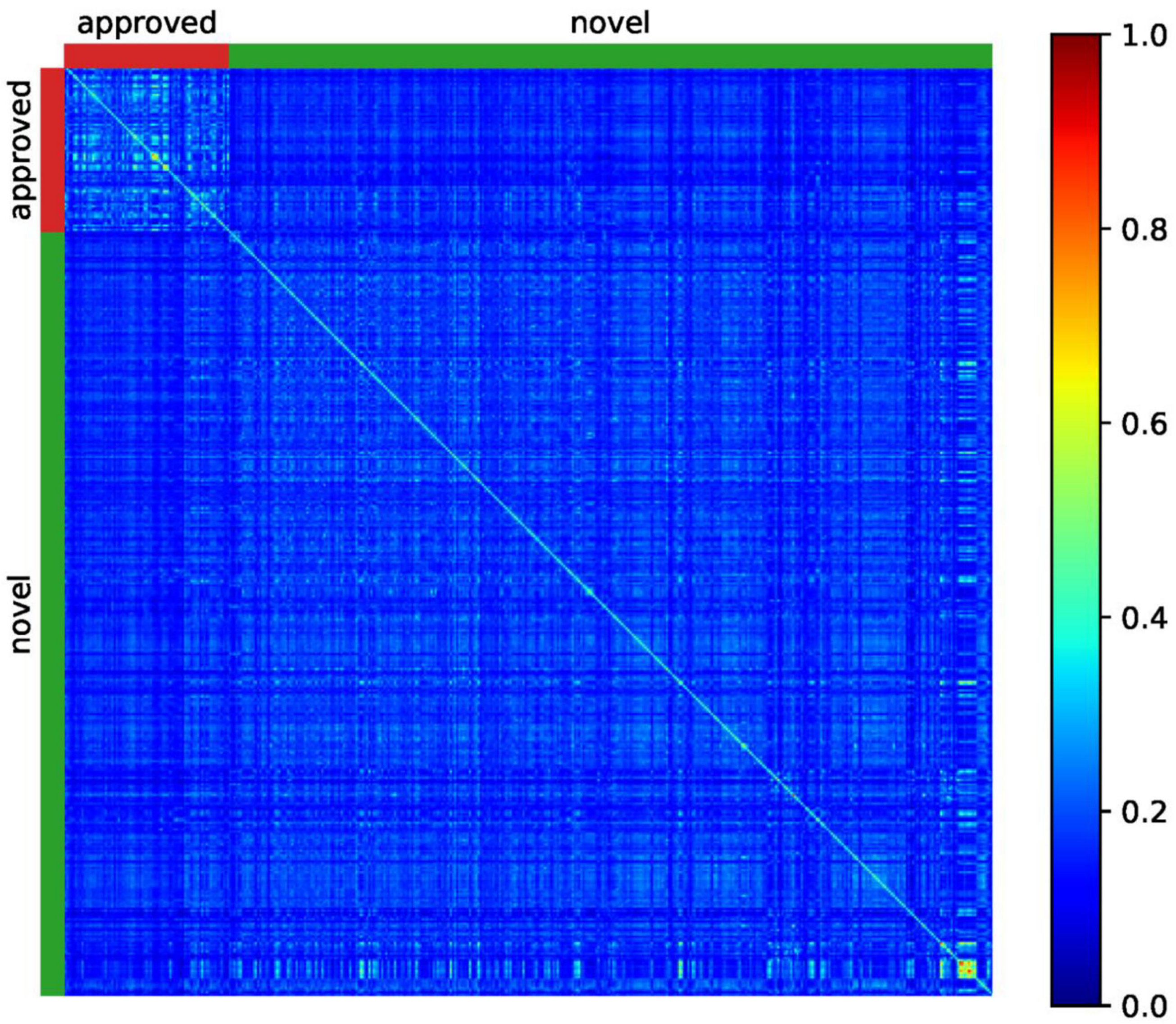

Supplementary Figure 6. FP2 molecular fingerprint similarity between FDA-approved antibacterial drugs and predicted antibacterial compounds. The color bar indicates the similarity from low (0) to high (1).

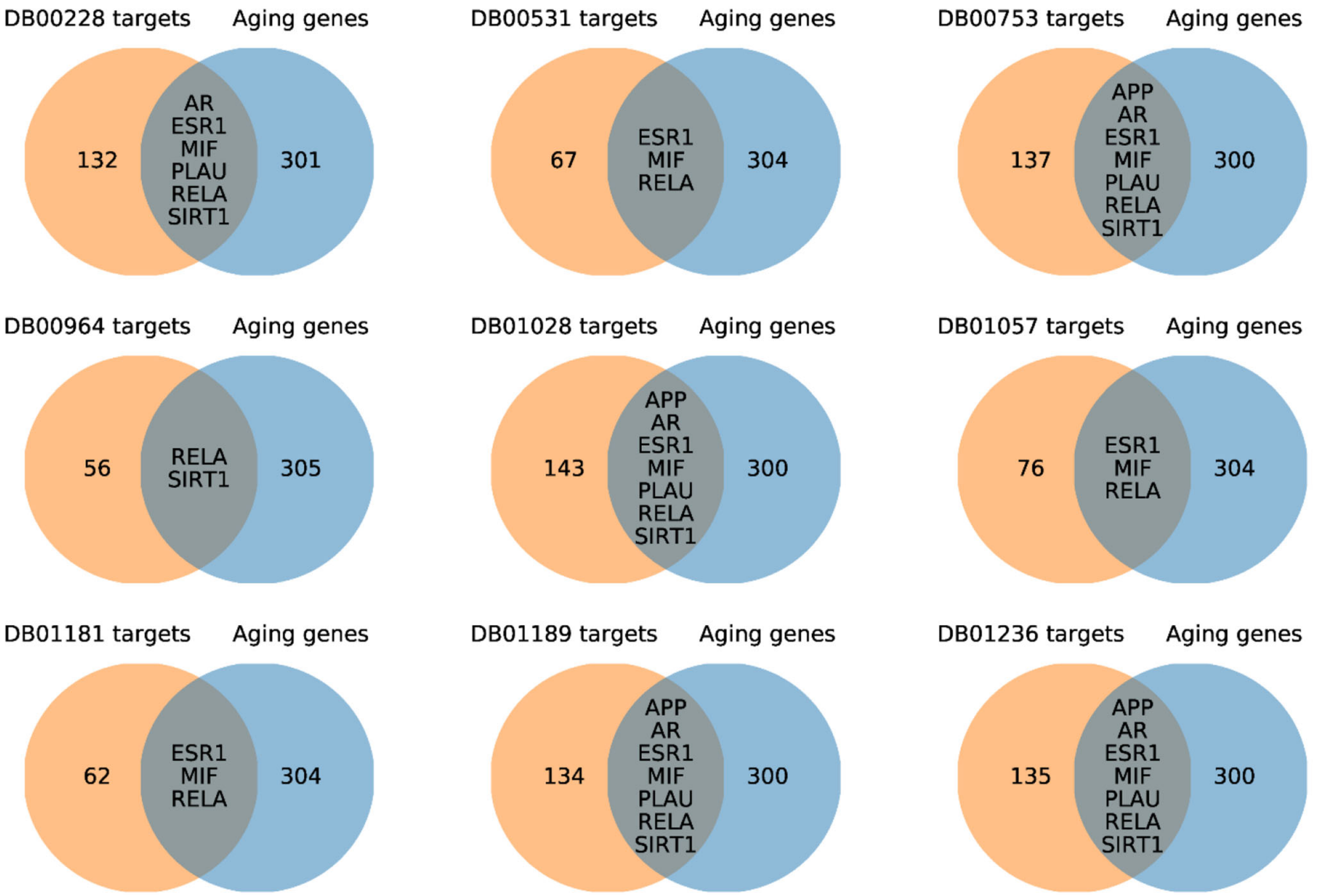

Supplementary Figure 7. Venn diagram of predicted drug targets and human aging-related genes. The union set of SEA, HitPick, and TargetNet predictions were chosen as target genes for the query drug. Human aging-related genes were downloaded from the Human Ageing Genomic Resources (HAGR).

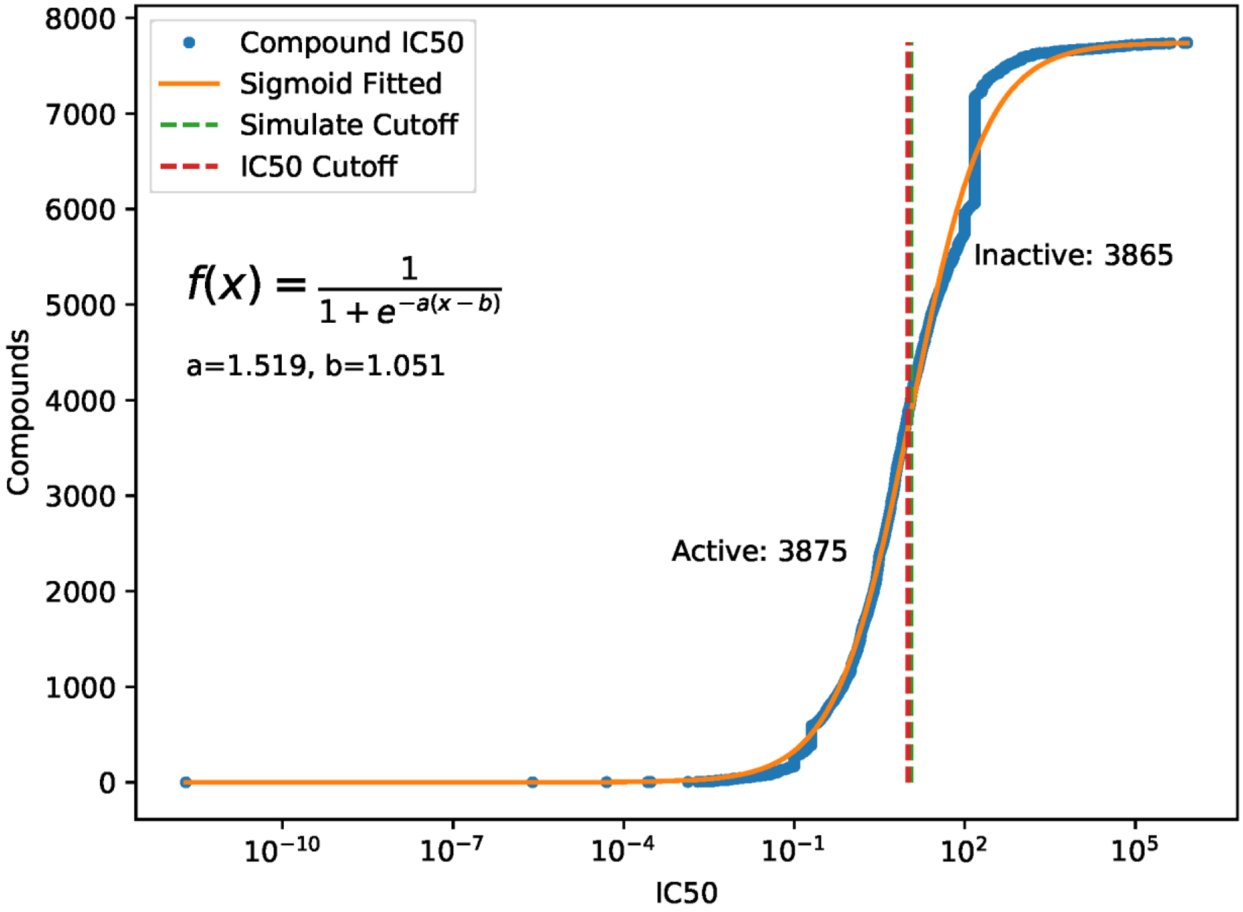

Supplementary Figure 8. Curve fitting of IC50 values of antibacterial compounds. The blue point indicates the IC50 values of all compounds. A sigmoid function was used to perform curve fitting and calculate the fitting parameters (yellow curve). The position of the maximum intercept of the function is defined as the fitting threshold (green dotted line), which is very close to the IC50 cutoff (10 µmol/L). Therefore, compounds with IC50 less than 10 µmol/L were defined as active antibacterial compounds, and those with IC50 higher than 10 µmol/L were considered inactive antibacterial compounds.
