# Supplementary material for: Screening of antibacterial compounds with novel structure from the FDA approved drugs using machine learning methods
Source: Aging (Albany NY). 2022 Feb 12;14(3):1448–72. doi: 10.18632/aging.203887 (PMC8876917; doi:10.18632/aging.203887)
Supplement: Supplementary Tables [file aging-14-203887-s002.pdf]

SUPPLEMENTARY TABLES

Supplementary Table 1. The prediction summary of different machine learning models.

Model	Number of predicted drugs	Number of overlapped drugs	*P*-value
SVM	1482	166	1.88E-8
RF	1539	170	2.45E-8
MLP	1398	153	4.21E-6
SVM, RF	1272	145	7.09E-7
SVM, MLP	1228	144	8.35E-8
RF, MLP	1162	137	2.56E-7
SVM, RF, MLP	1090	133	4.24E-8

Abbreviations: SVM: support vector machine; RF: random forest; MLP: multi-layer perception. *P*-values were calculated by the hypergeometric distribution model.

Supplementary Table 2. Core scaffolds and representative drugs of antibacterial compounds.

Categories	Core Structure	Represented	Name	Structure
Quinolones	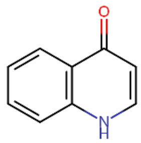	DB00218	Moxifloxacin	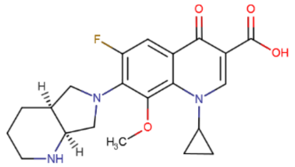
Penicillins	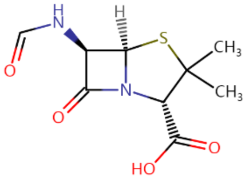	DB00578	Carbenicillin	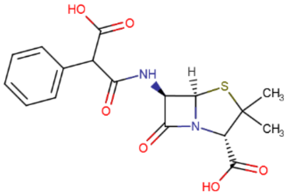
Oxazolidinones	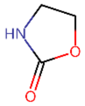	DB00601	Linezolid	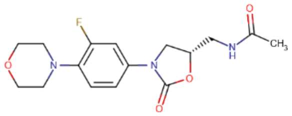
β-lactams	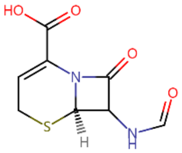	DB00689	Cephaloglycin	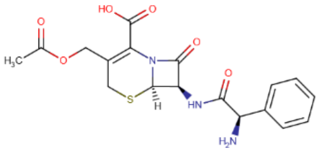
Sulfonamides	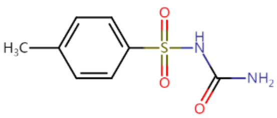	DB01124	Tolbutamide	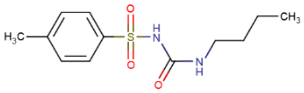
Lincosamides	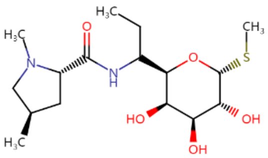	DB01190	Clindamycin	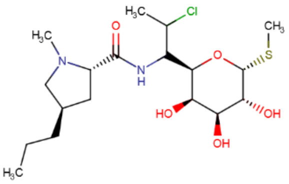

Carbapenems	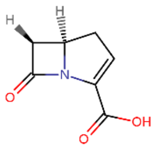	DB01598	Imipenem	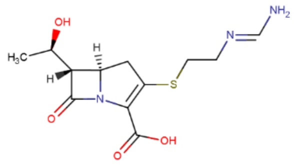
Cephalosporins	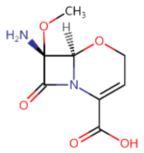	DB04570	Latamoxef	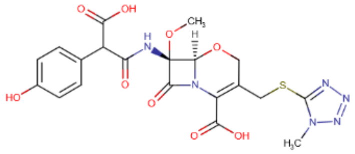

Supplementary Table 3. Novel predicted antibacterial drugs with high similarity to core scaffolds.

Categories	Predicted Drug	Query Size	Target Size	MCS Size	Tanimoto Coefficient	Overlap Coefficient
Quinolones	DB08820	11	29	11	0.38	1.00
Sulfonamides	DB00222	14	34	14	0.41	1.00
Sulfonamides	DB01016	14	33	14	0.42	1.00
Sulfonamides	DB01067	14	31	14	0.45	1.00
Sulfonamides	DB01251	14	37	14	0.38	1.00
Oxazolidinones	DB00315	6	21	6	0.29	1.00
Oxazolidinones	DB00660	6	16	6	0.38	1.00
Oxazolidinones	DB06228	6	29	6	0.21	1.00
Sulfonamides	DB00559	14	39	13	0.33	0.93
Sulfonamides	DB08439	14	26	13	0.48	0.93
Quinolones	DB00385	11	51	10	0.19	0.91
Quinolones	DB00445	11	39	10	0.25	0.91
Quinolones	DB00524	11	24	10	0.40	0.91
Quinolones	DB00670	11	26	10	0.37	0.91
Quinolones	DB00694	11	38	10	0.26	0.91
Quinolones	DB00695	11	21	10	0.45	0.91
Quinolones	DB00796	11	45	10	0.22	0.91
Quinolones	DB00904	11	22	10	0.43	0.91
Quinolones	DB00963	11	20	10	0.48	0.91
Quinolones	DB00997	11	39	10	0.25	0.91
Quinolones	DB01009	11	19	10	0.50	0.91
Quinolones	DB01022	11	33	10	0.29	0.91
Quinolones	DB01117	11	26	10	0.37	0.91
Quinolones	DB01148	11	29	10	0.33	0.91
Quinolones	DB01177	11	36	10	0.27	0.91
Quinolones	DB01204	11	32	10	0.30	0.91
Quinolones	DB01205	11	22	10	0.43	0.91
Quinolones	DB01325	11	18	10	0.53	0.91
Quinolones	DB01419	11	42	10	0.23	0.91

Quinolones	DB01698	11	43	10	0.23	0.91
Quinolones	DB02266	11	20	10	0.48	0.91
Quinolones	DB04880	11	17	10	0.56	0.91
Quinolones	DB05239	11	30	10	0.32	0.91
Quinolones	DB06193	11	24	10	0.40	0.91
Quinolones	DB06207	11	35	10	0.28	0.91
Quinolones	DB08822	11	42	10	0.23	0.91
Quinolones	DB08881	11	33	10	0.29	0.91
Quinolones	DB08911	11	37	10	0.26	0.91
Quinolones	DB08995	11	43	10	0.23	0.91
Quinolones	DB09079	11	40	10	0.24	0.91
Quinolones	DB09183	11	35	10	0.28	0.91
Quinolones	DB09214	11	19	10	0.50	0.91
Quinolones	DB11363	11	36	10	0.27	0.91
Quinolones	DB11577	11	28	10	0.34	0.91
Quinolones	DB11689	11	27	10	0.36	0.91
Quinolones	DB11699	11	21	10	0.45	0.91
Quinolones	DB11967	11	27	10	0.36	0.91
Quinolones	DB11986	11	41	10	0.24	0.91
Quinolones	DB13225	11	22	10	0.43	0.91
Quinolones	DB15477	11	30	10	0.32	0.91
Lincosamides	DB09419	24	10	9	0.36	0.90

Abbreviation: MCS: maximum common substructure. Tanimoto Coefficient = MCS Size/(Query Size + Target Size − MCS Size) Overlap Coefficient = MCS Size/min(Query Size, Target Size). The MCS algorithm was used to calculate structural similarities among small molecules. A total of 957 predicted novel antibacterial drugs were calculated among 8 core structures. The table showed 51 predicted drugs with an overlap coefficient >0.9 among 8 core structures, the results are sorted by overlap coefficient from high to low.

Supplementary Table 4. Details of the 9 predicted novel antibacterial drugs.

Drug ID	Name	Structure	Class	Indication
DB00228	Enflurane	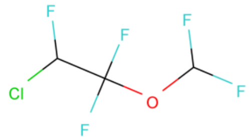	Organofluorides	Analgesia<br>General anesthesia
DB00531	Cyclophosphamide	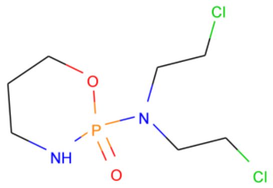	Organonitrogen compounds	Lymphoma<br>Multiple myeloma<br>Leukemia<br>Mycosis fungoides<br>Neuroblastoma<br>Ovarian adenocarcinoma<br>Retinoblastoma<br>Breast cancer

DB00753	Isoflurane	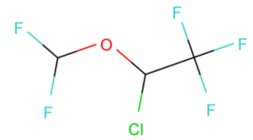	Organofluorides	General anesthesia
DB00964	Apraclonidine	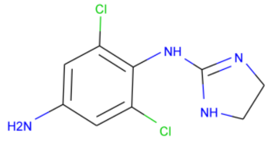	Benzene and substituted derivatives	Ocular hypertension<br>Postsurgical ocular hypertension
DB01028	Methoxyflurane	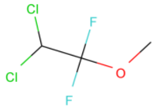	Organooxygen compounds	General anesthesia
DB01057	Echothiophate	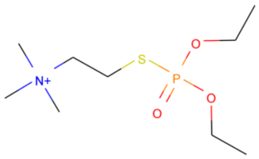	Organonitrogen compounds	Accommodative component in esotropia<br>Chronic angle-closure glaucoma<br>Open-angle glaucoma<br>Nonuveitic secondary glaucoma
DB01181	Ifosfamide	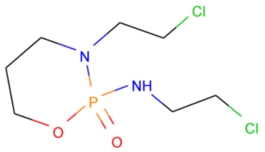	Oxazaphosphinanes	Germ cell testicular cancer<br>Cervical cancer<br>Soft tissue sarcomas<br>Osteosarcoma<br>Bladder cancer<br>Ovarian cancer<br>Small cell lung cancer<br>Non-Hodgkin's lymphoma
DB01189	Desflurane	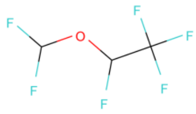	Organofluorides	General anesthesia<br>Maintenance of anesthesia therapy
DB01236	Sevoflurane	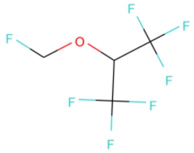	Organooxygen compounds	General anesthesia

Supplementary Table 5. Binary bits of different types of molecular fingerprints or vector features were used for machine learning modeling.

Compound Features	Description	Number of features
Molecular fingerprints		
FP2	FP2 Fingerprints	1024
FP3	FP3 Fingerprints	210
FP4	FP4 Fingerprints	307
DLFP	Daylight-like Fingerprints	2048
MACCS	MACCS keys	166
ECFP2	Extended-Connectivity Fingerprints, Iteration 1	1024
ECFP4	Extended-Connectivity Fingerprints, Iteration 2	1024

ECFP6	Extended-Connectivity Fingerprints, Iteration 3	1024
FCFP2	Functional-Class Fingerprints, Iteration 1	1024
FCFP4	Functional-Class Fingerprints, Iteration 2	1024
FCFP6	Functional-Class Fingerprints, Iteration 3	1024
Vector features		
mol2vec	Vector features based on Morgan fingerprints	200
SMILES2Vec	Vector features based on molecule SMILES	100
FP2VEC	Trainable embedding vectors based on fingerprints	100
